# Exploring the Expression and Function of cTyro3, a Candidate Zika Virus Receptor, in the Embryonic Chicken Brain and Inner Ear

**DOI:** 10.3390/v15010247

**Published:** 2023-01-15

**Authors:** Vashi Negi, Richard J. Kuhn, Donna M. Fekete

**Affiliations:** Department of Biological Sciences, Purdue Institute for Inflammation, Immunology and Infectious Disease, Purdue University, West Lafayette, IN 47906, USA

**Keywords:** Zika, Axl, Tyro3, otocyst, basilar papilla, neural tube

## Abstract

The transmembrane protein Axl was proposed as an entry receptor for Zika virus (ZIKV) infection in vitro, but conflicting results from in vivo studies have made it difficult to establish Axl as a physiologically relevant ZIKV receptor. Both the functional redundancy of receptors and the experimental model used can lead to variable results. Therefore, it can be informative to explore alternative animal models to analyze ZIKV receptor candidates as an aid in discovering antivirals. This study used chicken embryos to examine the role of chicken Tyro3 (cTyro3), the equivalent of human Axl. Results show that endogenous *cTyro3* mRNA expression overlaps with previously described hot spots of ZIKV infectivity in the brain and inner ear. We asked if ectopic expression or knockdown of cTyro3 influenced ZIKV infection in embryos. Tol2 vectors or replication-competent avian retroviruses were used in ovo to introduce full-length or truncated (presumed dominant-negative) cTyro3, respectively, into the neural tube on embryonic day two (E2). ZIKV was delivered to the brain 24 h later. cTyro3 manipulations did not alter ZIKV infection or cell death in the E5/E6 brain. Moreover, delivery of truncated cTyro3 variants to the E3 otocyst had no effect on inner ear formation on E6 or E10.

## 1. Introduction

Zika virus (ZIKV) is a mosquito-borne flavivirus that grabbed worldwide attention during the 2015–2016 outbreak that spread throughout the Americas. ZIKV preferentially infects neural progenitor cells and causes microcephaly in newborns [1,2,3,4,5] but can cause symptoms of varying severity from joint pains and flu-like illness [6,7,8] to Guillain–Barre Syndrome in adults as well [9,10,11]. With new and older clinical reports resurfacing, it was seen that similar to other flaviviruses, such as the Dengue virus (DENV), ZIKV had a much broader tissue tropism than previously suspected. ZIKV can cause loss of hearing [12,13,14] and vision [15,16], indicating minor symptoms may have gone unnoticed and were underreported prior to 2016. It is also found in bodily fluids [17] and can be sexually transmitted [18,19]. ZIKV preferentially targets tumorigenic glioblastoma stem cells (GSC), which is reversed upon inactivation of the GSC marker—Integrin α_v_β_5_ [20]. A study used genome-wide CRISPR screening to identify Integrin α_v_β_5_ as an internalization factor in GSCs [21]. Recently, the Neural Cell Adhesion Molecule NCAM1 was identified as a ZIKV receptor using proteomic-based studies and validated in human glioblastoma cells [22]. However, very little is known about the host receptors and attachment factors responsible for ZIKV infection in other permissive organisms and cell types due in part to functional redundancy in entry factors and conflicting reports from in vivo and cell culture studies [23]. This gap in knowledge has greatly hindered the development of therapeutic and preventive measures against ZIKV infection.

Some well-studied ZIKV receptor candidates include known flavivirus receptors such as dendritic cell-specific intercellular adhesion molecule-3 grabbing nonintegrin (DC-SIGN), heparan sulfate (HS), and the TIM (T-cell immunoglobulin and mucin domain) and TAM (Tyro3, Axl, Mertk) family of receptors that are expressed on dendritic cells and macrophages [24,25,26,27,28]. ZIKV can use the TIM1 receptor to infect the trophoblast layer of the placental barrier [29]. Unlike DENV and West Nile (WNV) flaviviruses that use the TIM1 receptor for infection as well [30] but do not cause microcephaly [31,32], ZIKV can efficiently use Axl to infect fetal endothelial cells by preferentially binding the Axl ligand Gas6 (Growth Arrest-Specific 6) [33,34]. In skin cells, Axl has been shown to have a greater effect on ZIKV entry than DC-SIGN, Tyro3, and TIM receptors [35]. Axl has also been implicated in Gas6-mediated ZIKV entry in human glial cells [36] but has shown no effect in human neural progenitor cells indicating that ZIKV uses multiple receptors in different cell types [37]. In vivo studies in immunocompromised mice showed no association between TAM receptors and ZIKV infection, although mouse Axl was shown to restore ZIKV infection in HeLa cells where human Axl was knocked out [38]. However, a major caveat of using the mouse model is that interferon (IFN)-α and -β responses in young mice, unlike in human newborns, are sufficient to fight ZIKV infection. Moreover, immunocompetent mice are not susceptible to ZIKV infection [39]. As a result, mouse models that eliminate the IFN response are useful for studying ZIKV infection, but they could also be masking important aspects of the IFN pathway in ZIKV pathogenesis. Therefore, not only is it important to find better methods to identify ZIKV receptors but also to establish an animal model that is infectable with ZIKV even in the context of an intact immune system, much like humans. In this study, our effort is directed toward understanding the link between TAM receptors and ZIKV infection in the embryonic chick model, given that ZIKV infection of chick neural progenitor cells has been established and likened to infection in humans in previous studies [40,41,42,43,44].

TAM receptors are an interesting category of possible ZIKV receptors since their expression patterns in various tissues and cells often match the tropism of ZIKV infection. TAMs belong to a distinct subfamily of receptor tyrosine kinases (RTK); they are highly conserved transmembrane proteins with diverse roles in innate immunity, vascular homeostasis, thrombus stabilization, and cancer progression [45]. During infection and Toll-like-receptor (TLR) engagement of antigen-presenting cells (APCs), TAMs are upregulated by type 1 IFN signaling and expressed on the surfaces of APCs. Only after T-cell activation by APCs, TAM ligands—Gas6 and Protein S (PROS1)—are made available to TAM RTKs [46]. This ensures that negative regulation of the innate immune response by TAM signaling only occurs as the adaptive immune system is being activated. After binding to their ligands, TAM RTKs dampen the type1 IFN response, the TLR-mediated response, and inflammation through the phosphorylation and activation of their tyrosine kinase intracellular domains [47]. They also play an important role in the clearance of apoptotic cells by phagocytic macrophages and dendritic cells that express them. TAM receptors sense the phosphatidylserine (PtdSer) lipids exposed on the outer membrane leaflets of apoptotic cells by activating scramblase XKR8 via their ligands [46]. APCs, such as dendritic cells and macrophages, with Gas6 or PROS1 ligands bound to TAM RTKs, can, in turn, bind PtdSer on apoptotic cells and phagocytose them [46]. However, in the absence of these receptors, they can be hyperactivated, resulting in autoimmune diseases [45,48]. Despite their important roles in the regulation of immune responses, TAM receptors are dispensable for embryonic development, as seen in TAM knockout mice [45] which makes them an attractive target for drug intervention.

Enveloped viruses can use these receptors for entry into host cells by binding to Gas6 or PROS1 (present in the bloodstream) through PtdSer lipids on their surfaces and thereby mimic apoptotic cells [47,48,49]. Flaviviruses, in particular, possess structural asymmetry and dynamic motion, which allows for exposure of the viral lipid layer and contributes to interaction with PtdSer receptors [50,51]. The advantages of this receptor interaction for the virus are two-fold: (1) it gains entry into APCs via clathrin-mediated endocytosis; (2) activated TAM signaling leads to the dampening of innate immune responses early in a viral infection before the adaptive immune system is primed for an attack [28,52,53,54]. For example, in human Sertoli cells, Axl is involved in both viral entry and regulation of ZIKV replication by modulating the immune responses of the cells [55]. TAMs can also play a post-entry role solely, as seen in WNV infection of HEK-293T cells and ZIKV infection of human astrocytes [56,57].

Despite the amount of research and effort dedicated to ZIKV studies since 2016, there is no vaccine or approved antiviral treatment available for ZIKV infection today. One critical bottleneck in the search for treatments is the lack of an ideal animal model to study ZIKV receptor and vaccine targets. While immunocompromised mouse models allow for easy genetic manipulation and faster screening, antiviral response to infection cannot be assessed accurately [58]. This has made it difficult to find targets that translate to clinical trials with human subjects as well [59]. Therefore, to investigate the role of TAM receptors in ZIKV entry, we have used the embryonic chick model as our in vivo system. This system offers easy manipulation of experimental factors, a low biohazard risk, and a medium-throughput analysis of the results [60]. We recently used this model organism to show that ZIKV can infect both the neural tube and inner ears [41], with permissivity of the latter tissue possibly relevant to an increased incidence of hearing loss following in utero exposure to ZIKV in humans [12,41]. Since the chick system lacks Axl, we turned our attention to chicken Tyro3 (cTyro3), which has high protein sequence homology with human Axl and major regions of the extracellular (receptor-binding) and intracellular (kinase) domains that are conserved (Figure S1A).

## 2. Materials and Methods

### 2.1. ZIKV Production and Titration

ZIKV strain, H/PF/2013, was grown in Vero (monkey kidney) cells at Richard Kuhn’s laboratory, Purdue University, and used for animal injections. This was provided as a passage 2 stock from Michael S. Diamond (Washington University School of Medicine, St. Louis, MO, USA). It was provided to the Diamond laboratory as a passage 3 stock from Xavier de Lamballerie (Emergence des Pathologies Virales, Aix-Marseille Université, Marseille, France) and the European Virus Archive Goes Global (EVAg). The virus stock was titered using a plaque assay [22] at 8.4 × 10^7^ PFU/mL suspension and stored at −80 °C until it was used for in ovo infections in chicken embryos.

### 2.2. Tissue Processing and Cryosectioning

For histological processing, whole embryos at embryonic day 2 (E2) to E4 or embryo heads (E5–E11) were fixed in 4% paraformaldehyde in phosphate-buffered saline (PBS). In the case of in situ hybridization experiments, fixation and all subsequent processing was maintained under RNase-free conditions. Fixed samples were dehydrated with graded sucrose (10%, 20%, 30% sucrose in PBS) and embedded in tissue freezing media (TFM, General Data Company, Cincinnati, OH, USA). The tissue blocks were frozen using liquid nitrogen and stored at −80 °C. For cryosectioning, 15 to 20 µm thick sections were collected on 3–4 sets of slides with alternate sections using a Leica^®^ CM1900 cryostat and stored in a −20 °C or −80 °C freezer.

### 2.3. RNAscope In Situ Hybridization

For *cTyro3* expression analysis, an mRNA probe against the cTyro3 transcript was used for RNAscope^TM^ in situ hybridization as per the manufacturer’s instructions (RNAscope^TM^ 2.5 HD Assay–RED Detection Kit (Chromogenic) User Manual by ACD). We used 26 normal embryos to do a time course of *cTyro3* expression in the head, with samples distributed as follows: E2 (n = 6), E3 (n = 6), E4 (n = 3), E5 (n = 2), E6 (n = 3), E8 (n = 2), E10 (n = 1) and E11 (n = 3). In situ hybridization was also performed on selected embryos subjected to experimental manipulations (such as plasmid electroporations and/or virus injections), with *cTyro3* expression evaluated on E5 (n = 16) or E10 (n = 1).

### 2.4. Plasmid Construct Design and Cloning

Plasmid constructs for cTyro3 overexpression and loss-of-function studies were synthesized by GenScript. We designed a Tol2-mediated bicistronic plasmid containing the coding sequences for cTyro3 and green fluorescence protein (GFP) genes separated by an internal ribosome entry site (IRES). When co-transfected with a Tol2 transposase plasmid, this allowed the full-length cTyro3 gene to be integrated into the host genome and transfected cells to be traced using GFP (Figure S3B). Stable overexpression of cTyro3 was confirmed in vitro in DF-1 chicken fibroblast cells (UMNSAH/DF-1 cell line; ATTC product #CRL-12203). A Tol2 plasmid expressing the GFP gene but lacking cTyro3 was used as a control for electroporation experiments.

Two truncated versions of cTyro3, one lacking the intracellular domain and the other lacking both the intracellular and transmembrane domains (Figure S3C), were cloned into the RCAS(A) avian retroviral vector. Both cTyro3 variants contained a C-terminal HA-tag. Plasmids were transfected into DF-1 cells to produce virus particles that were collected from the culture supernatant, concentrated by centrifugation, and titered on DF-1 cells by immunohistochemistry for viral gag protein with the 3C2 antibody (Developmental Studies Hybridoma Bank, DSHB, #AMV-3C2, AB_528098). Viral stock titers determined 42 h after infection were as follows: 7.35 × 10^8^ CFU/mL for RCAS(A)-cTyro3_Ex and 8.25 × 10^8^ CFU/mL for RCAS(A)-cTyro3_TM_Ex.

### 2.5. Plasmid Transfection and RCAS(A) Infection of DF-1 Cells

DF-1 cells were maintained at 37 °C in chick media containing Dulbecco’s Modified Eagle Medium (DMEM) (Gibco) supplemented with 10% Fetal Calf Serum (FCS), 1 mM L-glutamine, 1 mM Penicillin–Streptomycin, and 1% chick serum. In order to prepare samples for western blots of full-length cTyro3 protein expression, DF-1 cells were grown to sub-confluency in 6-well plates. Cells were then transfected with Tol-2 plasmid encoding cTyro3 and GFP genes using Lipofectamine 3000 reagent kit (Thermo Fisher Scientific, Waltham, MA, USA) according to manufacturer’s instructions and collected after 24 h. In order to prepare samples for expression of truncated HA-tagged cTyro3 proteins, cells were infected with concentrated RCAS(A) viral stocks and passaged until ~90–100% of the cells were immunopositive for expression of viral gag protein. Cells were dissociated and frozen, along with the culture supernatants. 

### 2.6. Western Blot

Samples transfected with Tol2 plasmids (expressing full-length cTyro3 and/or GFP) were lysed using 150 µL of 1X RIPA buffer (Santa Cruz #sc-24948) and 1 µL of 17.4 mg/mL PMSF (phenylmethylsulfonyl fluoride) protease inhibitor and rocked at 4 °C for 15 min. Cell lysate was collected and centrifuged at 10,000× *g* for 10 min at 4 °C. The pellet fraction was discarded, and the supernatant was analyzed on a 10% precast protein gel (Bio-Rad). Proteins were transferred onto a nitrocellulose membrane at 90 V for 100 min. Presence of protein was confirmed using Ponceau S staining followed by blocking with 3% BSA (bovine serum albumin). The membrane was probed using mouse PY20 anti-phosphotyrosine antibody (1:500, Santa Cruz, sc-508) and anti-GFP antibody (1:1000, rabbit IgG, Invitrogen, A11122). Infrared-labeled secondary antibodies (goat anti-mouse 680 and goat anti-rabbit 800, Licor) were used for visualization and imaging on an Odyssey CLx Near-Infrared Imaging System. The bands were identified as corresponding to PY20 and GFP based on their respective secondary antibodies used and their molecular weights and confirmed with individual antibody blots. The same membrane was then probed for mouse monoclonal anti-actin antibody (1:1000, Abcam, ab8226) and imaged again. Protein samples of cell lysates (prepared as explained above) and supernatants from RCAS-infected cell lines (expressing HA-tagged, truncated cTyro3) were transferred to nitrocellulose membranes and probed with mouse anti-HA.11 antibody (1:500, mouse IgG_1_, Covance, MMS-101P) and visualized with infrared secondary antibodies. The same membrane with the lysate samples was sequentially probed with rabbit anti-GAPDH antibody (1:1000, Abcam, ab9485). Western blot images were analyzed using Licor Image Studio software, Lincoln, NE, USA.

### 2.7. Animal Handling and Injections

For all experiments using chicken embryos, except those requiring RCAS injections, fresh fertilized White Leghorn chicken eggs were obtained from the Poultry department at Purdue University Animal Sciences Research and Education Center. The eggs were incubated at 38–39 °C and high humidity for normal development. For in ovo experiments, the eggs were windowed at E2 for staging using the Hamburger and Hamilton staging system [61]. The Tol2 and transposase plasmids were injected into the ventricles of the neural tube of E2 chick embryos at a molar ratio of 1:2 using a pulled glass capillary tube attached to a picospritzer and mounted on a micromanipulator. The plasmids were diluted in 0.25% fast green dye in a 1:10 (*v*/*v*) ratio such that the final concentration of DNA in the solution was ~3 µg/µL. Fast green dye was used for ease of visualization during injections for targeted delivery of the plasmids or the virus samples. The DNA was electroporated to the right side of the embryo using the equipment and methodology discussed previously [62]. The embryos were screened for GFP fluorescence in ovo at E3 using an epifluorescence dissection microscope to confirm plasmid expression. Another set of embryos was electroporated with fast green diluted in chick ringer solution (pH 7.4; 123.2 mM NaCl, 1.56 mM CaCl2, 4.96 mM KCl, 0.81 mM Na2HPO4 in water) as controls. 

Loss-of-function study was performed with RCAS(A)-cTyro3_TM_Ex and RCAS(A)-cTyro3_ Ex viral stocks. Specific-Pathogen-Free (SPF) eggs (purchased from Charles River Laboratories, Wilmington, MA, USA) received virus injections into the ventricles of the E2 neural tube. ZIKV was injected into the E3 midbrain ventricle and allowed two days for viral spread. The embryos were staged at every step and harvested at E5 or E6 for experiments pertaining to assessment of the embryonic brain. 

For inner ear experiments, both RCAS(A) viral vectors carrying cTyro3 truncated mutants—cTyro3_TM_Ex and cTyro3_Ex—were injected into the right otocyst at E3 and harvested at E6 or E10. In ovo manipulation and collection of chicken embryos were performed according to NIH guidelines and policies established by Purdue University Animal Care and Use Committee.

### 2.8. Immunohistochemistry

Slides carrying frozen tissue sections were thawed at room temperature and post-fixed in 4% paraformaldehyde for 10 min. High-protein blocking solutions (5% of goat or donkey serum or 2% bovine serum albumen, in PBS) were used to reduce non-specific antibody binding. The primary antibodies were diluted in blocking solution as follows: anti-GFP (1:1000, rabbit IgG, Invitrogen, Waltham, MA, USA, A11122), HA.11 (1:500, mouse IgG_1_, Covance, Princeton, NJ, USA, MMS-101P), dsRNA (1:500, J2, mouse IgG_2a_, SCICONS), 3C2 (1:5–1:40, mouse IgG_1_ hybridoma culture supernatant) [63], anti-HCS-1 (1:100, mouse IgG, BD Biosciences, San Jose, CA, USA), and axonal marker 3A10 (1:50, mouse IgG_1_, DSHB #3A10, AB_531874). Fluorophore-tagged secondary antibodies (Life Technologies, Carlsbad, CA, USA) were used at 1:250 or 1:500 dilution for fluorescence microscopy. For 3C2 and HCS-1/3A10 immunohistochemistry, diaminobenzidine (DAB) calorimetric staining was performed with a biotin-tagged secondary antibody and the ABC avidin–biotin–HRP enhancement step (Vector Laboratories, Newark, CA, USA), followed by diaminobenzidine oxidation reaction.

### 2.9. Imaging

Images of tissue sections were acquired using a Spot camera attached to a Nikon Eclipse E800 equipped with bright field, differential interference contrast, and epifluorescence or a Zeiss confocal microscope. Images were processed using ImageJ 1.47t (NIH open-source software, Bethesda, MD, USA). Images of immunostained DF-1 cells were taken with a Cytation7 (Agilent, Technologies, Santa Clara, CA, USA) multimode reader.

## 3. Results

### 3.1. In Situ Hybridization Indicates a Correlation between cTyro3 Expression and ZIKV Infection Hotspots

cTyro3, also known as Axl, is an 873 amino acid long membrane protein present on chromosome 5 (chr5:25149919-25189179 [64]) of the *Gallus gallus* genome [65,66]. Very little is known about the gene expression and localization of cTyro3 or other TAM receptors in the developing chick embryo (Geisha [67,68]). Since the TAM family of receptors is known to be highly conserved amongst vertebrate species in structure and function [69], we performed multiple sequence alignment of cTyro3 with human Axl (hAxl) and human Tyro3 (hTyro3) protein sequences using ESPript [70] to map similar and identical residues (Figure S1A). The extracellular region, composed of two N-terminal immunoglobulin-like and two fibronectin type III domains, consists of patches of identical residues. The N-terminus interacts with the Gas6 (or PROS1) ligand during TAM receptor activation leading to autophosphorylation of the tyrosine residues present in the kinase domain [71]. As expected, the C-terminal intracellular region containing the evolutionarily conserved kinase domain is highly similar amongst all three sequences, indicative of the conserved functions of these proteins. Overall, there is a ~43% sequence identity between cTyro3 and hAxl and ~69% between cTyro3 and hTyro3. There is no structural information available on cTyro3 functional domains; therefore, we relied on domain prediction data to identify various regions of the protein sequence (Figure S1B) [66]. Owing to the high sequence identity amongst these proteins, we reasoned that cTyro3 could be an Axl-substitute for viral entry in the chick system.

Studies performed in our lab using the embryonic chicken model have shown that the developing brain and inner ear are infectable with ZIKV [40,41]. Neural tubes infected at embryonic day two (E2) show “hotspots” of dsRNA (double-stranded RNA) signal indicating the presence of replicating ZIKV in neuroepithelial cells of the hypothalamus, midbrain–forebrain boundary, and the basal floor plate of the diencephalon. Signal was detected up to 7 days post-infection which is the median age of survival for infected embryos [40]. Infection of these hotspots and shrinking of the brain size (microcephaly) is observed as early as E5 [40]. If cTyro3 is involved in ZIKV entry, we reasoned that the mRNA should be present and the receptor protein expressed in ZIKV-susceptible tissues.

**Figure 1:**
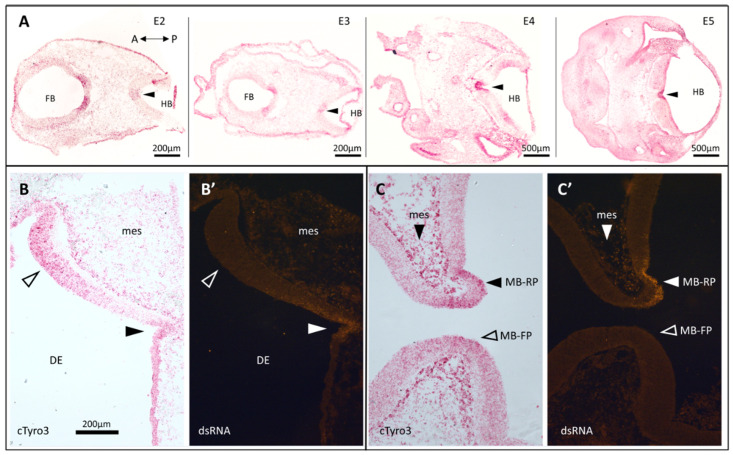
Endogenous cTyro3 mRNA expression pattern in the chick embryonic brain overlaps with known hotspots of ZIKV infection. (**A**) Sections from embryos sampled daily from E2 to E5 stained with *cTyro3* mRNA probe using RNAscope in situ hybridization (representative of n = 2–6 per day). *cTyro3* expression is strong in regions known to be readily infected with ZIKV, including the floor plate of the hindbrain (closed arrowheads). *cTyro3* mRNA expression of a ZIKV-infected E5 embryonic chick brain in (**B**) the diencephalon and (**C**) the midbrain shows regions with overlapping ZIKV infection in alternate sections (**B’**) and (**C’**) respectively (closed arrowheads). Surrounding regions that did not show overlap are indicated with open arrowheads. Mesenchyme surrounding the brain, previously shown to be permissive for ZIKV, strongly expresses *cTyro3* and, in the ZIKV-infected embryo of panel (**C’**), is positive for dsRNA. Abbreviations: A, anterior; DE, diencephalon; FB, forebrain; HB, hindbrain; MB-FP, midbrain floor plate; MB-RP, midbrain roof plate; mes, mesenchyme; P, posterior.

There are no commercially available antibodies against cTyro3; therefore, we used an anti-sense probe to map mRNA expression patterns using RNAscope in situ hybridization (ISH) [72] on paraformaldehyde-fixed tissue sections of control chick embryos. Our results show that *cTyro3* expression is not uniform and that specific regions of the brain (Figure 1) have higher levels of expression. Interestingly, these regions overlap with the following known ZIKV infection hotspots previously reported for the brain [40]: the midbrain and hindbrain floor plates (Figure 1A—closed arrowheads; Figure S2), the diencephalon (Figure 1B and Figure S2), and the midbrain roof-plate (Figure 1C—closed arrow; Figure S2). Images of full cross-sections through the head show the consistency of these patterns and also show high expression in the developing retina (Figure S2), which is also permissive for ZIKV infection. Conversely, the dorsal regions of the midbrain and hindbrain that show relatively low *cTyro3* mRNA expression are also known to be less frequently infected with ZIKV [40]. We confirmed the overlap of higher *cTyro3* signal and ZIKV infectivity in the brain and mesenchyme of E5 embryos injected with ZIKV on E2 (Figure 1B,C—closed arrowheads and closed arrows) while noting that not all *cTyro3* hot spots are infected (Figure 1B,C—open arrowheads) and areas with lower *cTyro3* expression levels could also be infected.

**Figure 2:**
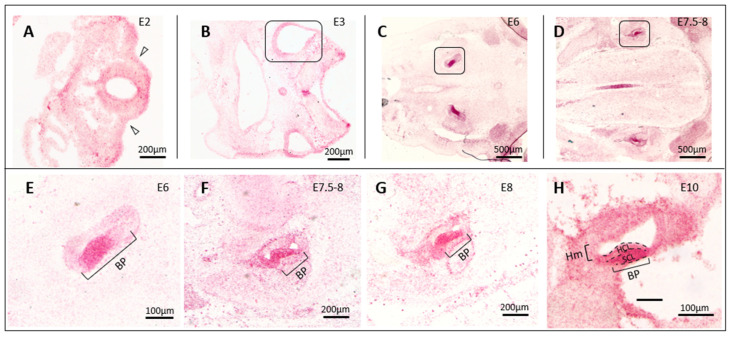
Endogenous cTyro3 mRNA expression levels in the embryonic chick inner ear. (**A**–**H**) *cTyro3* mRNA expression in the inner ear was visualized at E2–E6, E7.5, E8 and E10–11 (n = 1 to 6 per specified day). Expression level is low at (**A**) E2 when the otic cup invaginates (open arrowheads) but increases with age (**B**–**D**) as the inner ear sensory organs begin to form. Positive staining is particularly robust in the anlage of the basilar papilla, the auditory sensory organ in birds (boxed regions at E6 and E7.5–8). (**E**–**H**) The sensory region of the basilar papilla (BP) has exceptionally high *cTyro3* mRNA expression from E6-E10. (**H**) The supporting cell layer of the BP has somewhat higher *cTyro3* mRNA expression than the hair cell layer at E10. Abbreviations: BP, basilar papilla; E, embryonic day; Hm, non-sensory homogene cells; HCL, hair cell layer; SCL, supporting cell layer.

In studying the brain expression of *cTyro3,* we noticed strong signals in the developing inner ear. The epithelium of the inner ear develops from the placode-derived otocyst, while the surrounding loose mesenchyme and cartilaginous otic capsule are mesoderm-derived. ZIKV injection into the chicken otocyst on E2-E5 leads to infections of all regions of the inner ear labyrinth when evaluated 2–8 days later [41]. The otic mesenchyme was also found to be susceptible to ZIKV infection [41]. During the time window of ZIKV susceptibility, the developing inner ear expresses moderate to high levels of *cTyro3* (Figure 2). The sensory region of the embryonic basilar papilla, the organ responsible for hearing in the bird, shows exceptionally high expression of *cTyro3* at E6, with signal readily identified in progenitor cells, until E11 when the hair cells are mid-differentiation (Figure 2E–H). The otic mesenchyme also expresses *cTyro3* (Figure 2F–H).

### 3.2. Ectopic cTyro3 Overexpression Does Not Increase ZIKV Infection beyond the Usual Hotspots of the Embryonic Brain

**Figure 3:**
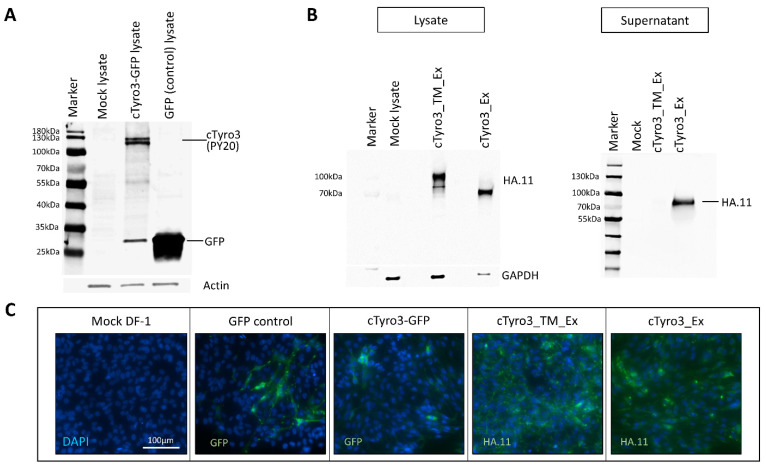
cTyro3 constructs successfully express expected proteins. Western blot analysis of transfected lysates and supernatants in DF-1 cells to confirm protein expression of (**A**) full-length cTyro3 and (**B**) truncated HA-tagged cTyro3 mutants. (**A**) Full-length cTyro3 has intact kinase activity as shown by PY20 antibody signal that detects phosphorylated tyrosine residues only in cells transfected with the cTyro3 plasmid. GFP is present in both the cTyro3-GFP and control GFP transfected lysates. (**C**) Transfected cells were fixed and immunostained for GFP or HA.11 antibodies to confirm protein expression in cells. For these and other images with GFP labeling, the endogenous GFP fluorescence signals were enhanced by immunostaining for the protein using a secondary antibody with green fluorescence. Scale bar = 100 µm. Abbreviations: kDa, kiloDaltons.

The process of viral entry in host cells involves sequential stages of attachment, receptor-binding, internalization, and post-internalization or uncoating events mediated by the interaction of the virion with various host proteins [73]. TAM receptors are thought to be involved in the attachment and entry of enveloped viruses but may also facilitate infection at a post-entry stage by enhancing replication or antagonizing Type I IFN responses via their kinase domains [28,56,74,75]. Therefore, cTyro3 could be involved in various possible capacities during ZIKV infection of chick embryos, as shown schematically in Figure S3A. In order to test the function of cTyro3 in the context of embryonic development and/or ZIKV infection, constructs for either overexpression (Figure S3B) or loss-of-function studies (Figure S3C) were designed to introduce cTyro3 and its kinase mutants into the chick embryos. For overexpression, a Tol2-mediated plasmid containing the full-length cTyro3 and green fluorescence protein (GFP) genes was generated by Genscript and transfected into DF-1 cells. Another Tol-2 plasmid without the cTyro3 gene was used as a GFP control. A PY20 antibody (sc-508) that detects phosphorylated tyrosine (p-Tyr) was used in a western blot to confirm the presence of activated cTyro3 in lysates of DF-1 embryonic chicken fibroblasts taken 24 h after they were transfected with the Tol2-cTyro3 plasmid (Figure 3A). Transfection of cTyro3 plasmid yielded two protein bands at expected sizes above 100 kDa, corresponding to the mature and partially glycosylated forms of the protein [76]. GFP expression was confirmed in the transfected lysate (Figure 3A) and in fixed transfected cells (Figure 3C) using a GFP antibody.

**Figure 4:**
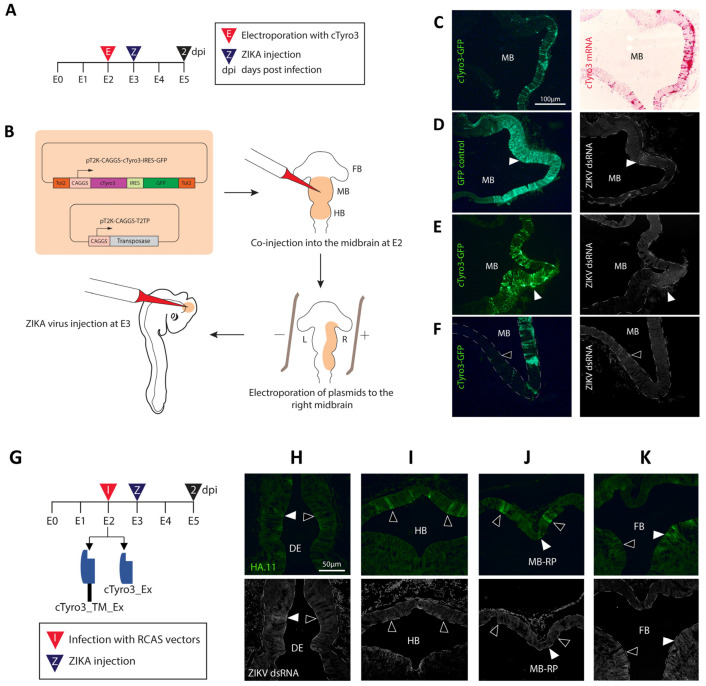
Ectopic cTyro3 overexpression or truncation does not correlate with the locations of ZIKV infection. (**A**) Timeline of the experiment to test the effect of cTyro3 overexpression on ZIKV infection in the embryonic brain. (**B**) Experimental methodology for expressing plasmids encoding bicistronic mRNA to encode full-length cTyro3 and GFP proteins, and designed for stable integration and expression in chick embryos using a transposase expression plasmid. Both plasmids are electroporated to the right side of the neural tube at E2 (black triangle, 2 dpi) followed by ZIKV injection into the midbrain ventricle 24 h later (blue triangle, Z). (**C**–**F**) Analysis of ZIKV infection in the presence of ectopic cTyro3 from 18 embryos. (**C**) Co-localization of GFP protein and *cTyro3* mRNA after electroporation was confirmed using immunohistochemistry and RNAscope in situ hybridization, respectively, on alternate sections at E5. Note how high *cTyro3* over-expression appears in the dorsal midbrain in comparison to endogenous expression levels. (**D**–**F**) A dsRNA antibody was used to localize ZIKV infection in the red channel (shown in grey scale). ZIKV infection did not consistently overlap with ectopic cTyro3 transfection (visualized by GFP in the green channel) as overlap could be seen for both (**D**) the control (n = 1/5 embryos) as well as (**E**) the experimental samples (n = 3 embryos). (**F**) Regions of the midbrain that did not have any ectopic cTyro3 also showed ZIKV infection (open arrowheads) while cTyro3-transfected cells (green) nearby had no infection (n = 12 embryos). Scale bar = 100 µm. (**G**) Timeline of experiment to test the effect of cTyro3 dominant-negative mutants on ZIKV infection. RCAS viral vectors encoding the truncated cTyro3 mutants were injected into the E2 neural tube (black triangle, 2 dpi), followed by ZIKV injection one day later (blue triangle, Z). (**H**–**K**) Known hotspots of ZIKV infection were visualized for reduction in infection compared to RCAS control embryos. ZIKV infection levels, already relatively low in these experiments, did not appear to be blocked in areas transfected with cTyro3 mutant constructs. Open arrowheads show HA.11-positive regions with no corresponding ZIKV infection while closed arrowheads show regions with ZIKV infection despite expressing a dominant-negative receptor. Images are representative of trends seen for n = 8 embryos screened for the cTyro3_TM_Ex mutant and n = 6 embryos screened for the cTyro3_Ex mutant. Scale bar = 50 µm. Abbreviations: DE, diencephalon; E, embryonic day; FB, forebrain; HB, hindbrain; MB, midbrain; MB-RP, midbrain roof plate.

The cTyro3-GFP or control GFP plasmids, along with a plasmid containing a Tol2 transposase gene, were introduced into the E2 brain ventricles of chick embryos, specifically in the midbrain (Figure 4A,B). E2 in chicken is comparable to the fourth week in human gestation (Carnegie Stages 11 and 12) [77] when the neurons of the brain and eye primordia are still undifferentiated. The plasmids were electroporated into the right brain such that the left un-electroporated side would serve as the contralateral control. The efficiency of co-transfection of these plasmids and subsequent stable expression of GFP/cTyro3 was first confirmed in DF-1 cells using GFP antibodies. One of the requisites for a viral receptor candidate to be established as a biologically significant entry receptor is its ability to render non-permissive cells permissive to infection. These cells must not express said receptor candidate endogenously and only become infected when the receptor candidate is introduced via genetic transfer [78]. The dorsal midbrain is a region with consistently low or no ZIKV infection in chicken embryos at E3–E5, compared to the floor plate of the midbrain that shows high ZIKV susceptibility [40]. As mentioned above, the dorsal midbrain is also a region with low endogenous *cTyro3* expression (Figure 2B). To ask whether cTyro3 would render this region more permissive to ZIKV infection, we injected ZIKV into the midbrain ventricle 24 h after electroporation of the full-length cTyro3 constructs. Embryos were allowed two days for viral spread, then harvested at E5 and evaluated for spatial overlap between ectopic cTyro3 expression and ZIKV presence (Figure 4C–F).

We selected embryos that showed ectopic expression of cTyro3 in the right dorsal midbrain at E5, as confirmed by *cTyro3* in situ hybridization and/or immunofluorescence with GFP antibody in alternate sections (Figure 4C). GFP-stained sections were co-stained with dsRNA antibodies to detect ZIKV (Figure 4D–F). ZIKV was present in the usual hotspots (midbrain floor plate and basal plate) as expected, although infection levels overall were modest due to limitations in the virus titer. Overlap between GFP and dsRNA signals was not consistent or exclusive to cTyro3-electroporated embryos, indicating that infection in the midbrain could not be attributed exclusively to ectopic cTyro3 expression. The overexpression of cTyro3, under our experimental conditions, did not affect overall ZIKV infection levels in the embryonic brain, suggesting that it is not a limiting factor for ZIKV infection. We also investigated the effect of cTyro3 overexpression on macrophage levels in the embryonic brain using a colony-stimulating factor 1 receptor (CSF1R) marker. E2 embryos were electroporated, as shown in Figure 4B, and harvested E5-E6 brain tissue samples were stained for macrophage marker CSF1R. While no differences were observed in the midbrain (Figure S4B,B’), the CSF1R signal on the electroporated (right) side was slightly higher in and around the forebrain region than that on the (control) left side of the embryos (Figure S4C,C’,D,D’).

Thawani and colleagues (2018) showed that ZIKV-infected cells in the chick embryonic brain had increased cell death at 3 days post-infection (dpi), after which viral load drops [40]. Previous studies have shown that resident macrophages of the brain, the microglia, are responsible for TAM-dependent clearance of dead cells by phagocytosis and of stressed-but-viable neurons by phagoptosis in adult mice [79,80] and disruption of TAM-kinase signaling prevents TAM-mediated phagocytosis [81]. To test whether the higher CSF1R signal seen in cTyro3-electroporated samples could be involved in the clearance of cells undergoing cell death, we stained these sections with a cell death marker, activated Caspase 3 (Casp3). However, no change in the Casp3 signal was observed.

### 3.3. Expression of Truncated Versions of cTyro3 Does Not Reduce ZIKV Infection in the Embryonic Brain

For loss-of-function studies, two constructs were designed in replication-competent retroviral vector RCAS(A) [82] that expressed either the extracellular and transmembrane domains [cTyro3_TM_Ex] or only the extracellular domain (soluble form) of cTyro3 [cTyro3_Ex] with C-terminal hemagglutinin (HA) tags (Figure S3C). Expression of the truncated proteins was confirmed by western blot analysis of the lysates and supernatants of DF-1 cells 24 h after RCAS(A) infection using the HA.11 antibody (Figure 3B). Infected DF-1 cells were fixed and stained with the HA.11 antibody to confirm protein expression (Figure 3C). For in ovo experiments, the RCAS viral vectors were injected into the brain ventricles at E2, ZIKV was delivered on E3, and embryos were incubated for two more days to allow for ZIKV replication to make dsRNA (Figure 4G). The presence of the cTyro3 mutant proteins was verified by immunostaining for the HA.11 antibody. ZIKV infection was localized in fixed sections of E5 heads from controls (GFP virus) and experimental (mutant cTyro3 virus) using dsRNA immunostaining. There was no appreciable and consistent reduction in the level of infection in the usual hotspots of ZIKV infection, indicating that the presence of dominant-negative mutants of cTyro3 alone did not reduce ZIKV infection levels (Figure 4H–K and Figure S5A–E). ZIKV infection was seen in the diencephalon (Figure 4H and Figure S5D), the midbrain roof-plate (Figure 4J and Figure S5B), and the basal plate of the forebrain (Figure 4K and Figure S5E) despite the presence of the cTyro3 dominant negative. However, this evaluation was hampered by high sample variability and the fact that viral infection was low in and around ZIKV hotspots for a majority of the samples. Embryos infected with RCAS(A) vectors carrying the cTyro3 dominant-negative mutants [cTyro3_TM_Ex (n = 3) and cTyro3_Ex (n = 5)] but not infected with ZIKV were also tested for changes in cell-proliferation levels using a phosphohistone H3 (pH3) antibody. However, no obvious difference was seen in cell–proliferation.

### 3.4. Infection of the Inner Ear with cTyro3 Dominant-Negative Viruses Does Not Affect Cell Death or Hair Cell Differentiation

**Figure 5:**
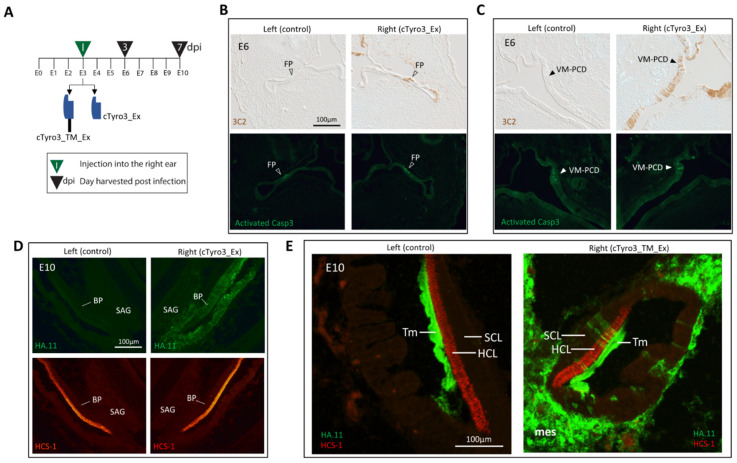
Expression of cTyro3 dominant negatives does not affect cell death and hair cell expression in the inner ear. (**A**) Timeline of experiment. ZIKV was injected on E3 (green triangle) with embryos processed on E3 or E10 (black triangles) (**B**–**E**) Infection of the right inner ears with RCAS(A) is indicated by immunostaining of viral gag protein with 3C2. Infection does not change immunostaining for activated Caspase 3 in (**B**) the fusion plate (FP) of the lateral canal (open arrowheads) or the (**C**) ventromedial hotspot of programmed cell death (VM-PCD) (closed arrowheads), when compared with the same regions in the uninfected left ear. (**D**,**E**) Expression of the hair cell marker HCS-1 is unchanged on the infected right side compared to the uninfected left side. In (**E**), the tectorial membrane is non-specifically labelled with HA.11 (green) antibody in both ears. Scale bar = 100 µm. Abbreviations: BP, basilar papilla; dpi, days post-infection; FP, canal fusion plate; HCL, hair cell layer; SAG, statoacoustic ganglion; SCL, supporting cell layer; Tm, tectorial membrane; VM-PCD, ventromedial hotspot of programmed cell death.

High expression levels of *cTyro3* in the embryonic inner ear suggest that this RTK has a function in normal ear development, irrespective of any role in ZIKV infection. Inner ear morphogenesis involves changing patterns of cell proliferation and apoptosis as the simple otic vesicle grows and gives rise to the complex labyrinth of chambers, ducts, and sensory organs that subserve hearing and balance [83,84]. Given its role in the clearance of dying cells, we looked for evidence that *cTyro3* might be involved in programmed cell death as the ear develops. The focal regions where cell death is high and reproducible in the inner ear epithelium were selected for evaluation. The largest and most prolonged region of cell death is the ventromedial wall adjacent to the nascent auditory ganglion at E4–E7 [83], although its function remains unknown. Other prominent foci of cell death during embryonic inner ear development are located in the anlage of the three semicircular canals when fusion plates transiently appear at E6. Semicircular canal morphogenesis of the chick is stalled by experimentally reducing cell death in the fusion plates [83]. To explore the role of cTyro3 in the context of inner ear development, we attempted to knock down the cTyro3 function in ovo. RCAS(A) viruses encoding either the kinase mutant cTyro3_TM_Ex (Figure S3C) or the soluble form of the protein cTyro3_Ex (Figure S3D) were injected into the right otocyst at E3, with the left side serving as a within-animal control (Figure 5A). On E6, the locations of known foci of cell death in the inner ear were screened for the presence of RCAS infection on the right side using 3C2 and/or HA.11 immunostaining. Embryos with adequate infection at the relevant locations on the right side were then evaluated for left-right differences in the presence of apoptotic cells (visualized as phase-bright foci by differential interference microscopy) or in the levels of activated-Casp3 immunostaining. Our prediction was that the mutant variants of cTyro3 might abrogate the clearance of dying cells and possibly alter the morphogenesis of the inner ear. For inner ears infected with cTyro3_TM_Ex, no overt differences were seen in the location or amount of apoptosis or activated caspase-3 in the fusion plates (n = 10) or the ventromedial region of programmed cell death (VM-PCD) (n = 7). The same observation was made for fusion plates (n = 5) and the VM-PCD (n = 4) in embryos infected with cTyro3_Ex (Figure 5B,C). In summary, none of the RCAS-infected inner ears had left-right differences in semicircular canal formation or overall morphogenesis.

Our in situ hybridization analysis showed that the sensory region of the basilar papilla was one of the strongest domains of *cTyro3* expression in cross-sections through the head during the second week of embryogenesis (Figure 2B). Around this time, between E5 and E9, hair cells and supporting cells that are derived from common progenitors pull out of the division [85]. The pseudostratified sensory epithelium then organizes into an apical layer of hair cells that reside above the cell bodies of supporting cells, whose apical processes separate adjacent hair cells from one another. Individual supporting cells span the entire depth of the epithelium. Shortly after becoming post-mitotic, hair cells begin to express hair-cell-specific markers. Therefore, we also assessed the effect of cTyro3 dominant-negatives on hair cell formation at E10, one week after RCAS viral vectors were injected into the right otocyst, using hair cell markers HCS-1 or myosin VI. To be included in the analysis, alternate sections were screened for virus-mediated transgene delivery in the right basilar papilla (and also often in the adjacent periotic mesenchyme), as indicated by the presence of 3C2-positive and/or HA.11-positive cell clusters. Neither the differentiation nor the distribution of hair cells was affected within the infected clusters in comparison either to nearby uninfected cells on the same side or to the basilar papilla on the opposite side. This includes sensory organs infected with either cTyro3_Ex (Figure 5D; n = 5) or cTyro3_TM_Ex (Figure 5E; n = 5).

## 4. Discussion

While many flaviviruses, such as WNV and DENV, can cause severe neurological defects, only ZIKV amongst them has been associated with microcephaly in newborns [31,32,86]. This difference in disease phenotype can be explained by a difference in receptor candidacy that affects its neural tissue tropism [33]. Hence, vaccines and antivirals against ZIKV should target specific receptor interactions that are linked to microcephaly and other ZIKV-associated neurological malformations. Viruses can target the process of TIM/TAM-mediated removal of apoptotic cells for entry in a phenomenon called “apoptotic mimicry” [47,87]. The TAM receptor Axl has been shown to be an entry factor for ZIKV in human fetal endothelial cells [33]. ZIKV has been shown to enter liposomes expressing PS and PE (phosphatidylethanolamine) lipids that bind to the Axl ligand Gas6 [88]. In immunocompetent mice, ZIKV infection has been directly linked to increased expression levels of Gas6 and subsequent downregulation of Type 1 IFN response [89]. Gas6 levels were also higher in ZIKV-infected human patients suffering from neurological complications being treated in Brazil compared to patients that had ZIKV infection but no neurological complications [89]. This highlights the relevance of Gas6-TAM-receptor-related studies in finding vaccines and antivirals for ZIKV-induced neurological disorders.

Previous studies have shown that Axl is a candidate entry receptor for ZIKV in specific human cell lines—radial glial cells, skin cells, Sertoli cells, fetal and adult endothelial cells, astrocytes, microglia, glioblastoma cells and neural stem cells (based on expression analysis) [33,34,35,36,55,57,75,90]. However, Axl knockdown or knockout failed to show an effect on ZIKV infection in neural progenitor cells in cerebral organoids, TAM knockout mice, and primary genital epithelial cells [37,91,92]. Hastings et al. (2017) speculated that it is possible that human cell lines may not express the full spectrum of ZIKV receptors present in human cells in vivo [38], and thus, it is of significant interest to find new or modify existing in vivo models to better mimic the human in vivo system.

While no in vivo animal model can be 100% identical to humans, the embryonic chick system has the advantage of displaying an intact immune system, as compared to immunocompromised mice, and this advantage can highlight the effects of Type 1 INF responses during ZIKV infection, especially as it pertains to TAM signaling. TAM receptors also function as mediators of innate immune responses and provide negative feedback during inflammation [47]. Thus, deliberately modifying the IFN signaling in mouse models could affect downstream Axl activation in ZIKV-receptor studies. The vast similarities between the human and avian immune systems have also been discussed extensively in previous reviews [93,94,95,96,97]. Wild birds can be naturally infected with ZIKV [98], and chick embryos tested in laboratory research are shown to be susceptible to ZIKV infection displaying neurological defects similar to those observed in infected newborns [40,42]. Although little is known about the function and expression patterns of TAM receptors and their ligands in the chick embryonic system, cTyro3 shares high sequence similarity with both hAxl and hTyro3, especially in the intracellular kinase domain responsible for downstream signaling (Figure S1). Our mRNA expression analysis of *cTyro3*, Axl equivalent in chick embryos, shows that this TAM receptor is present in known hotspots of ZIKV infection in the brain and the inner ear. From embryonic day E2-5, *cTyro3* mRNA expression is higher in the midbrain roof plate and floor plate and in the ventral regions of the hindbrain and midbrain compared to the dorsal regions (Figure 1A and Figure 2A–D), which positively correlates with increased incidence of ZIKV infection [40]. The sensory region of the basilar papilla in the chicken inner ear is responsive to ZIKV infection and also shows a robust expression of *cTyro3* mRNA as early as E6 in development (Figure 2E). Although ZIKV infection of the retina is not discussed in this study, it is another ZIKV hotspot with strong *cTyro3* mRNA expression (Figure S2). Therefore, the chick embryonic system has the potential to be a promising animal model for studying ZIKV receptor candidates in vivo, and more studies are needed to exploit this potential.

In order to further investigate the correlation between cTyro3 mRNA expression and ZIKV infection in the chick embryonic brain, we attempted to overexpress the full-length protein along with GFP on the right side of the brain using electroporation of a Tol2 plasmid carrying these genes accompanied by its transposase plasmid at E2. This was performed to ensure that cTyro3-GFP genes were integrated into the chick genome and expressed throughout the time window of ZIKV infection from E3–E5. Due to the lack of an antibody against cTyro3, we relied on RNAscope in situ hybridization to confirm the overlap of *cTyro3* mRNA and GFP protein at E5 and subsequently used the GFP antibody to identify regions transfected with cTyro3 plasmid. Since Axl is activated by Gas6 and not PROS1 [71], the chick equivalent of Gas6 (cGas6) is presumably needed in the embryos for cTyro3 to be activated and interact with ZIKV. While no information is available on the expression of cGas6 in chick embryos, a study in rat embryos showed that *Gas6* is expressed in various cells of the central nervous system as early as E14 [99]. Considering the conserved nature of the Gas6 protein function in vertebrates, we can expect cGas6 to follow a similar expression timeline across species. This aligns well with the timeline of our experiment since E14 in rats corresponds to approximately E3.75 in chickens [77]. Furthermore, we used a p-Tyr antibody (PY20) to test whether DF-1 cells (a chicken embryo fibroblast line) transfected with the cTyro3-GFP plasmid contained phosphorylated cTyro3 at its expected size (Figure 3A) when analyzed on a western blot membrane. We observed two bands in the DF-1 cTyro3-GFP cell lysate above 100 kDa that could not be seen in the DF-1 cell lysate containing only the GFP protein. This suggests that the cTyro3 protein expressed in DF-1 cells using the Tol2 plasmid has phosphorylated tyrosine and is functionally active. Any signal corresponding to endogenous cTyro3 in DF-1 cells is likely very low since bands of the same size for p-Tyr in mock and control GFP lysates are hard to see. Thus, the exogenous cTyro3 protein encoded by the Tol2 plasmid is capable of being phosphorylated to become functionally active if its ligand, possibly Gas6, is present in chick embryos.

Using immunohistochemistry, we analyzed ZIKV infection of electroporated and controlled E5 embryos using a ZIKV dsRNA antibody for fluorescence imaging. The goal was to determine whether ectopic cTyro3 expression (indicated by GFP fluorescence) made the midbrain, specifically the dorsal region with its lower endogenous *cTyro3* transcripts, more permissive to ZIKV infection. Our results showed that while some embryos with good cTyro3 overexpression throughout the right midbrain did have ZIKV infection in the dorsal region, the dsRNA signal was not present throughout the GFP-positive (cTyro3 transfected) neuroepithelium (Figure 4E). Such overlap in a small part of the right dorsal midbrain was also seen in an embryo electroporated with a GFP-control plasmid (Figure 4D). The majority of the embryos did not show any overlap of dsRNA and GFP signals in the midbrain, suggesting that the presence of cTyro3 alone is not enough to enhance ZIKV infection of the neuroepithelium at E5. However, higher titer ZIKV stocks might be needed to overcome these negative findings. We also observed higher ZIKV infection levels in the mesenchyme surrounding the right side of the midbrain, overexpressing cTyro3 in 8 out of 18 embryos; however, this was also true for some embryos that failed to overexpress cTyro3 ectopically after electroporation (n = 4/11 embryos).

TAM signaling is important for maintaining immune homeostasis via the regulation of innate immune responses and the clearance of apoptotic cells by macrophages [100]. These processes are linked to viral infections, and it is interesting to ask whether manipulation of cTyro3 changes macrophage activation or cell death clearance in chicken embryos. TAM receptors are known to be involved in macrophage activation and polarization through the phosphoinositide 3 kinase (PI3K)/Akt pathway [101,102]. Hence, we also tested for macrophage localization near sites in ectopic cTyro3 expression in or around the brain using a CSF1R marker. We observed slightly higher levels of macrophages on the right side of the electroporated embryo in the mesenchyme near the forebrain and the retina but not the midbrain (Figure S4). However, there was no overlap between the CSF1R and GFP (cTyro3) stains, so it is not obvious if this phenotype is a result of cTyro3 signaling.

Our loss-of-function studies also showed that expressing HA-tagged cTyro3 mutants lacking the kinase domain using the RCAS viral vector did not have an effect on the overall ZIKV infection levels in the E5 embryonic brain (Figure 4H–K) and could not be correlated with the mutant expression. Embryos with good overall ZIKV and RCAS infection in known ZIKV hotspots of the midbrain (basal and roof plates) and the diencephalon showed regions of overlap, suggesting that the mutant protein was unable to bind to ZIKV particles to prevent entry through a functional receptor present nearby. Another possible role for cTyro3, illustrated schematically in Figure S3, is as an attachment factor for ZIKV. For this role, the intracellular kinase domain would not be required, and the cTyro3 mutant would instead assist in infection. However, we did not see an overall increase in ZIKV infection of HA.11-positive cells compared to the surrounding HA.11-negative cells. For instance, 4 out of 12 embryos tested had much more ZIKV infection on one side of the brain in the mesenchyme, perhaps due to the angle at which the virus was injected into an E3 brain, but had similar levels of HA.11 stain on both sides. However, this analysis was hindered by relatively low ZIKV infection levels in these embryos and high sample variability.

The role of TAM receptors in chick embryonic development is not well understood. However, the consistently high level of *cTyro3* mRNA expression in the sensory region of the inner ear epithelium and its surrounding mesenchyme is noteworthy. In an attempt to understand the role of cTyro3 in inner ear development, we examined the effect of expressing the functionally inactive kinase mutant on cell death at E6 and hair cell differentiation at E10 (Figure 5). We infected the right ear of E3 embryos with RCAS vectors encoding mutant proteins to look for alterations in inner ear development. However, we did not observe a phenotype either in the cell death of prominent apoptotic foci at E6 or in hair-cell differentiation in the basilar papilla at E10. Thus, the function of cTyro3 in otocyst development, particularly in the differentiating basilar papilla, remains unknown.

This study provides new information on the localization of cTyro3, a poorly understood protein, in the chicken embryo. We also used the embryonic chick as an in vivo model for ZIKV-receptor studies. However, our conclusions on the role of cTyro3 in rendering neural progenitors permissive to ZIKV were tempered by relatively low levels of infection obtained in ovo with the viral titers used in this analysis. Moreover, due to the lack of cTyro3 and cGas6 antibodies and the absence of structural information for these proteins, our study was limited in its ability to answer questions pertaining to the exact role of cTyro3 in viral infections and embryonic development. Studies using humanized cTyro3 receptor or transgenic hAxl supplemented with hGas6 in the chick system could prove beneficial in exploiting the advantages of this in vivo model in studying the role of Axl in ZIKV entry. Further studies are needed to answer these questions and further establish the embryonic chick system as a useful model to explore ZIKV receptor candidates.

## Data Availability

The data presented in this study are available on request from the corresponding author. The data are not publicly available due to complexity of the image data.

## Supplementary Materials

The following supporting information can be downloaded at: https://www.mdpi.com/article/10.3390/v15010247/s1, Figure S1: Multiple sequence alignment and predicted domains of cTyro3 protein [7]; Figure S2: *cTyro3* mRNA expression across the E5 chick embryonic brain; Figure S3: cTyro3 construct designs for overexpression and loss-of-function studies; Figure S4: Ectopic cTyro3 overexpression can lead to increase in macrophage expression in the E5 forebrain and mesenchyme; Figure S5: Expression of cTyro3_Ex extracellular domain in the E5 chick embryonic brain does not correlate with locations of ZIKV infection.
