# Supplementary material for: Exploring the Expression and Function of cTyro3, a Candidate Zika Virus Receptor, in the Embryonic Chicken Brain and Inner Ear
Source: Viruses. 2023 Jan 15;15(1):247. doi: 10.3390/v15010247 (PMC9867072; doi:10.3390/v15010247)
Supplement: Supplementary file 1 [file viruses-15-00247-s001.zip › viruses-2133360-supplementary.pdf]

Supplementary figures

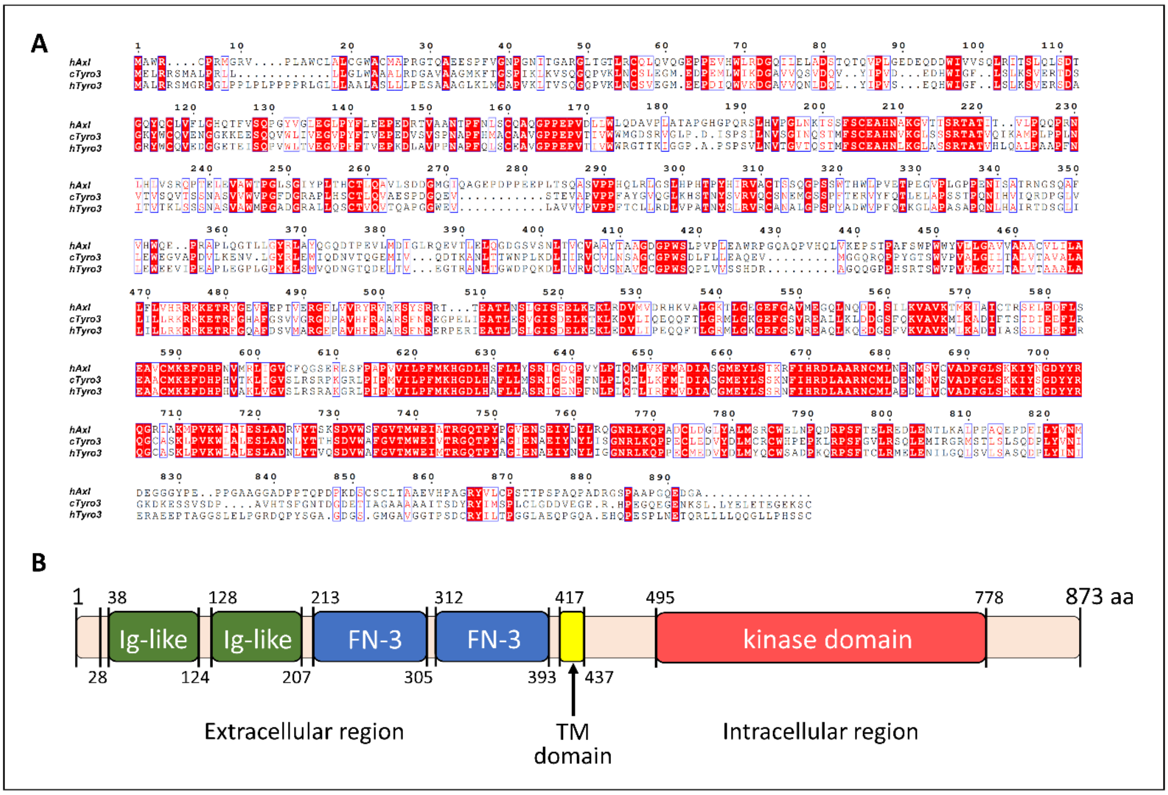

Supplementary figure S1

Multiple sequence alignment and predicted domains of cTyro3 protein. (A) A comparison of the amino acid sequences of cTyro3 (uniprot ID:Q98949), human Axl (hAxl; uniprot ID: P30530) and human Tyro3 (hTyro3; uniprot ID:Q06418) proteins using ESPript [7]. Residues conserved in all three sequences are shown in red highlighted boxes, while residues conserved in any two are shown in red font inside a blue box. Missing residues are indicated by black dots. cTyro3 has ~43% sequence identity with hAxl and ~69% with hTyro3 with majority of the C-terminus cytoplasmic region conserved. (B) cTyro3 predicted domains (from NCBI database-Tyro3 *gallus gallus* NP_989958.2) – a 28 amino acid signal peptide at the start of the protein precursor is cleaved to give cTyro3 protein with two Immunoglobulin-like (Ig-like) and two Fibronectin type III (FN-3) domains in the extracellular region and an intracellular kinase domain separated by a transmembrane (TM) domain as shown.

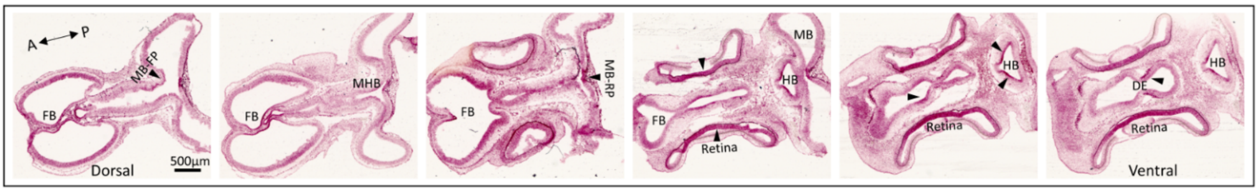

Supplementary figure S2

***cTyro3* mRNA expression across the E5 chick embryonic brain.** An E5 embryo stained with *cTyro3* mRNA probe using RNAscope *in situ* hybridization. Expression levels are higher for specific regions of the midbrain, hindbrain, retina and diencephalon compared to the surrounding tissue; some of these regions are indicated by black arrowheads. Scale bar = 500µm.

Abbreviations: DE, diencephalon; FB, forebrain; HB, hindbrain; MB-FP, midbrain floor plate; MB-RP, midbrain roof-plate; MHB, MB-HB boundary.

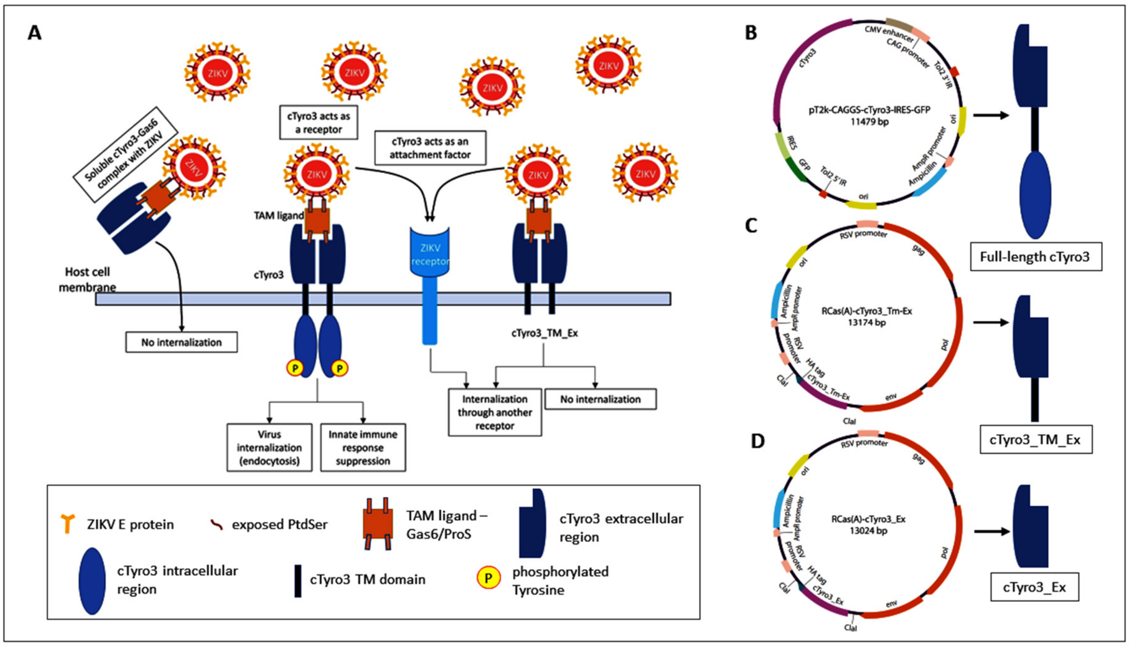

Supplementary figure S3

cTyro3 construct designs for overexpression and loss-of-function studies. (A) Schematic showing possible roles of cTyro3 in ZIKV entry. If cTyro3 is a *bona fide* ZIKV receptor, it will bind its ligand (Gas6) which in turn is bound to phosphatidylserine (PtdSer) lipids on the virus surface. A virus particle will be internalized via endocytosis mediated by the kinase domain, as predicted previously for hAxl, leading to productive infection. No internalization would occur in the absence of the kinase domain and the virus particle would remain bound to the extracellular domain of the protein on the cell surface. Soluble cTyro3-Gas6 complex would trap ZIKV particles in the extracellular space preventing entry. In another scenario, cTyro3 could act as an attachment factor and lead to infection in a kinase-independent manner. (B-D) Based on the domain predictions in S1(B) and the scenarios in S2(A), three constructs were designed to express (B) the full-length cTyro3 using Tol2-mediated gene transfer, and (C) cTyro3 lacking the kinase domain (TM_Ex) or (D) with extracellular domain (Ex) alone using avian retrovirus vector RCAS(A). These RCAS vectors utilize a splice donor site at the start of the gag gene, and a splice acceptor site (not shown) in front of the cTyro3 transgenes to transcribe a separate mRNA used for transgene protein translation. Plasmid maps of the constructs and their expected protein expression are shown.

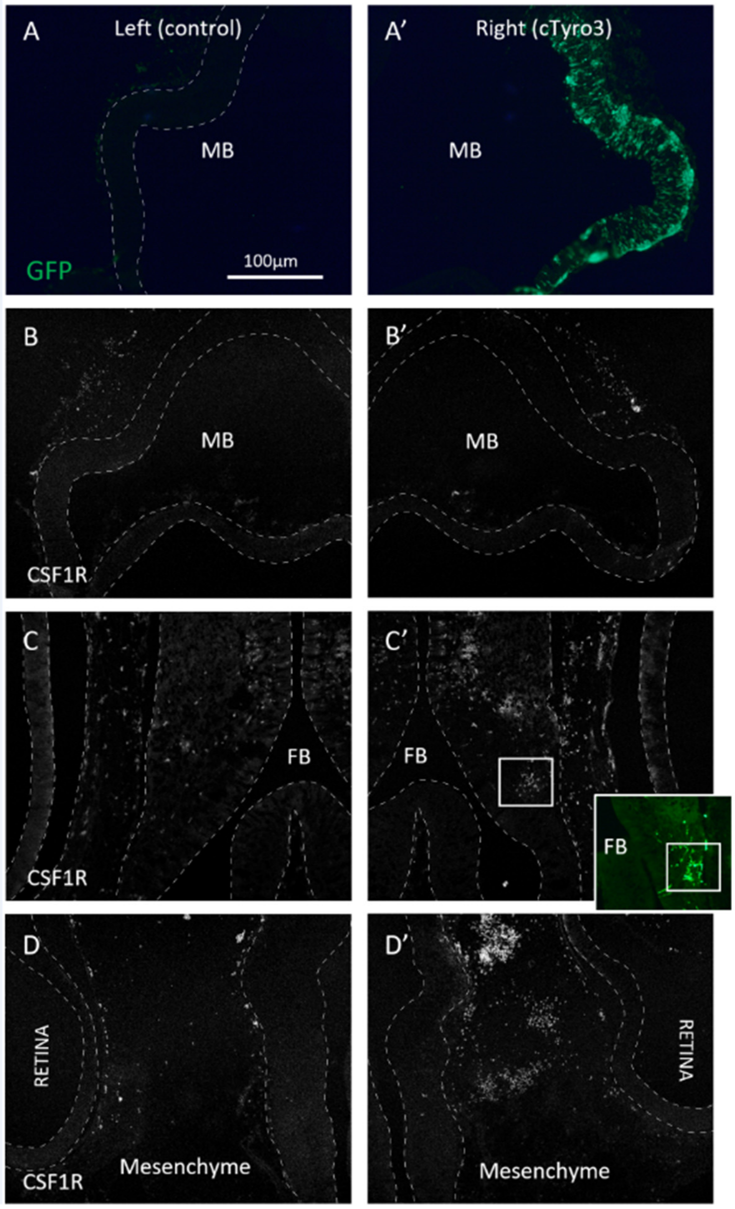

Supplementary figure S4

Ectopic cTyro3 overexpression can lead to increase in macrophage expression in the E5 forebrain and mesenchyme. GFP expression in the (A) left (contralateral control) and the (A') right cTyro3 electroporated side of the midbrain. *cTyro3* overexpression does not change macrophage (CSF1R) expression in the (B, B') midbrain but leads to slight increase in the (C, C') forebrain and the (D, D') surrounding mesenchyme. Images are representative of n=2 embryos. Scale bar = 100µm.

Abbreviations: FB, forebrain; MB, midbrain.

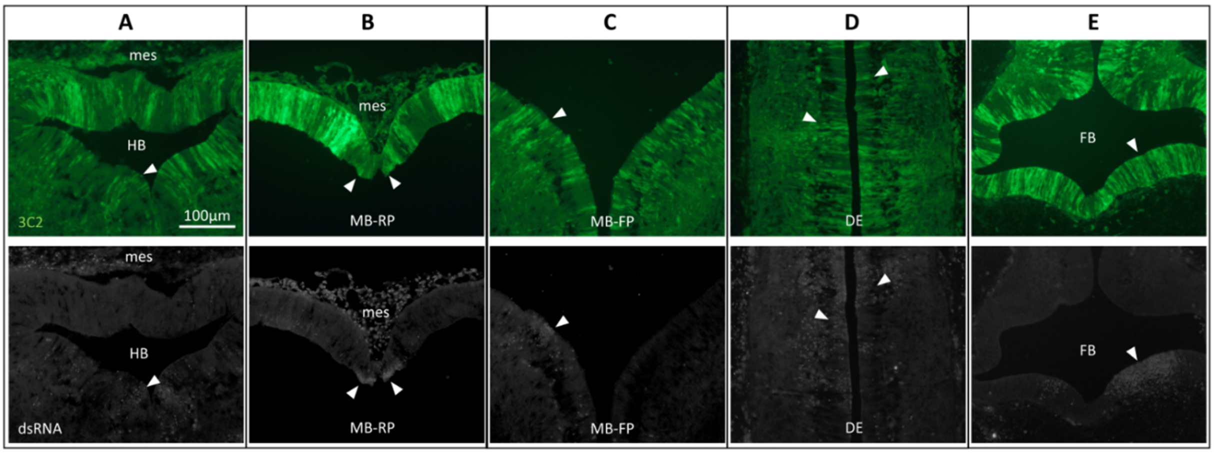

Supplementary figure S5

Expression of cTyro3_Ex extracellular domain in the E5 embryonic chick brain does not correlate with locations of ZIKV infection. Embryos infected with RCAS(A)-cTyro3_Ex at E2 and with ZIKV at E3 were analyzed for reduction in ZIKV infection in known hotspots. RCAS infection, visualized using 3C2 antibody, did not correlate with ZIKV dsRNA signal in the (A) hindbrain (n=3/5), (B, C) midbrain (n=4/10), (D) diencephalon (n=4/9) and (E) the forebrain (n=4/9). Closed arrowheads indicate regions with overlapping 3C2 and dsRNA signals.

Abbreviations: DE, diencephalon; E, embryonic day; FB, forebrain; HB, hindbrain; MB-FP, midbrain floor plate; MB-RP, midbrain roof plate; mes, mesenchyme.
